# Two Duplicated *Ptpn6* Homeologs Cooperatively and Negatively Regulate RLR-Mediated IFN Response in Hexaploid Gibel Carp

**DOI:** 10.3389/fimmu.2021.780667

**Published:** 2021-11-26

**Authors:** Jin-Feng Tong, Li Zhou, Shun Li, Long-Feng Lu, Zhuo-Cong Li, Zhi Li, Rui-Hai Gan, Cheng-Yan Mou, Qi-Ya Zhang, Zhong-Wei Wang, Xiao-Juan Zhang, Yang Wang, Jian-Fang Gui

**Affiliations:** ^1^ State Key Laboratory of Freshwater Ecology and Biotechnology, Institute of Hydrobiology, The Innovative Academy of Seed Design, Chinese Academy of Sciences, Wuhan, China; ^2^ College of Life Sciences, University of Chinese Academy of Sciences, Beijing, China; ^3^ Hubei Hongshan Laboratory, Chinese Academy of Sciences, Wuhan, China; ^4^ Fisheries Institute, Sichuan Academy of Agricultural Sciences, Chengdu, China

**Keywords:** Gibel carp, SHP1, negative regulator, autophagy, MITA, interferon

## Abstract

Src homology region 2 domain-containing phosphatase 1 (SHP1), encoded by the *protein tyrosine phosphatase nonreceptor type 6* (*ptpn6*) gene, belongs to the family of protein tyrosine phosphatases (PTPs) and participates in multiple signaling pathways of immune cells. However, the mechanism of SHP1 in regulating fish immunity is largely unknown. In this study, we first identified two gibel carp (*Carassius gibelio*) *ptpn6* homeologs (*Cgptpn6-A* and *Cgptpn6-B*), each of which had three alleles with high identities. Then, relative to *Cgptpn6-B*, dominant expression in adult tissues and higher upregulated expression of *Cgptpn6-A* induced by polyinosinic-polycytidylic acid (poly I:C), poly deoxyadenylic-deoxythymidylic (dA:dT) acid and spring viremia of carp virus (SVCV) were uncovered. Finally, we demonstrated that *Cg*SHP1-A (encoded by the *Cgptpn6-A* gene) and *Cg*SHP1-B (encoded by the *Cgptpn6-B* gene) act as negative regulators of the RIG-I-like receptor (RLR)-mediated interferon (IFN) response *via* two mechanisms: the inhibition of *Ca*TBK1-induced phosphorylation of *Ca*MITA shared by *Cg*SHP1-A and *Cg*SHP1-B, and the autophagic degradation of *Ca*MITA exclusively by *Cg*SHP1-A. Meanwhile, the data support that *Cg*SHP1-A and *Cg*SHP1-B have sub-functionalized and that *Cg*SHP1-A overwhelmingly dominates *Cg*SHP1-B in the process of RLR-mediated IFN response. The current study not only sheds light on the regulative mechanism of SHP1 in fish immunity, but also provides a typical case of duplicated gene evolutionary fates.

## Introduction

SHP1, which is encoded by the *ptpn6* gene, is a member of the family of PTPs that catalyze the dephosphorylation of tyrosyl residues in proteins phosphorylated by protein tyrosine kinases (PTKs) (1, 2). PTPs and PTKs participate in cell-cycle progression, cell motility and invasion, as well as in cell death and apoptosis (3, 4). SHP1 has two N-terminal SH2 domains (N-SH2 and C-SH2), followed by a classical PTP domain and a C-terminal tail (C-tail), and contains two tyrosyl phosphorylation sites. In response to the activation signal, SHP1 is recruited to membrane-bound inhibitory receptors through the binding of its SH2 domain to tyrosine-phosphorylated immunoreceptor tyrosine-based inhibitory motifs (ITIMs) (5, 6). It is a critical regulator of immune cell development and function and has been considered as an immune checkpoint (1). Studies on natural mouse mutants (motheaten and motheatenviable) and conditional cell-type-specific Shp1 mutants (Ptpn6f/fCD19Cre/+) both showed that Shp1 plays critical roles in regulating the differentiation and/or activation of B cells (7, 8), T cells (9), dendritic cells (10), and neutrophils (11). Moreover, it is required to establish life-long protective humoral immunity (8). Once recruited to the inhibitory receptors through ITIMs, SHP1 controls multiple signaling pathways to obtain an ideal immune response (12–16).

Although the roles of SHP1 have been well documented in mammals (17, 18), the research in fish is still scarce. Several studies have demonstrated that SHP1 can be recruited by different immune-type receptors, such as channel catfish (*Ictalurus punctatus*) leukocyte immune-type receptors (IpLITRs) (19, 20), sea lamprey (*Petromyzon marinus*) T-cell receptor-like molecule (TCRL) (21) and gibel carp diverse immunoglobulin domain-containing protein (DICP) (22). In addition, only a few studies have reported the expression changes of SHP1 and its role in the immune reaction after bacterial challenge (23, 24). Morpholino knockdown of *ptpn6* in zebrafish embryo was shown to lead to the hyperinduction of innate immune response genes such as *ifnφ1*, *il1b*, *il8*, *tnfa*, and *tnfb* during *Salmonella typhimurium* or *Mycobacterium marinum* infection (23). The expression of *ptpn6* in Nile tilapia was upregulated after *Streptococcus agalactiae* infection and may involve in the B cell receptor (BCR) signaling pathway (24). However, to date, little is known about the function of fish SHP1 in regulating IFN mechanism.

Most extant vertebrates have evolved from polyploid ancestors. It is now universally accepted that two rounds (2R) of whole-genome duplication (WGD) occurred at the root of vertebrates, and a subsequent teleost fish-specific (Ts3R) WGD took place after the divergence of tetrapods and teleosts (25–30). More recent WGD events have also occurred in some actinopteriygiian families, including Acipenseridae, Cyprinidae, and Salmonidae (31, 32). In general, WGD are thought to increase genetic complexity and variability, which would in turn give rise to evolutionary novelties and broader adaptabilities (28, 33). During the subsequent post-polyploid diploidization (PPD), the duplicated genes experience divergent evolutionary trajectories and undergo partitioning under relaxed purification options. Their evolutionary fates include retention/loss, non-(pseudogenization), sub- or neo-functionalization (33, 34). Although the evolutionary fates of duplicated genes have been well elaborated in plant polyploids, only a few studies on recurrent animal polyploids have been reported due to the difficulties in discriminating the different homeologs/alleles of duplicated genes. We had recently elaborated the divergent functions of duplicated *foxl2* and *viperin* homeologs in gibel carp (35, 36). However, no single model or ideal could explain all evolutionary ways and fates of duplicate genes (30, 33, 37). Therefore, it is necessary to investigate more cases to deepen our understanding of the evolutionary “rules” in animal polyploids.

Gibel carp, which is widely distributed across the Eurasian continent (38–43), has been recognized as an evolutional hexaploid with over 150 chromosomes in comparison with tetraploid goldfish (*C. auratus*) with 100 chromosomes (44–46). Analyses of several conserved genes suggests that two extra rounds of polyploidy, an early allopolyploidy and a later autopolyploidy, had taken place during gibel carp evolution (35, 36, 47–50). Disease resistance breeding has become an important hotspot in the current research landscape. In our previous study, we identified several candidate resistant-related genes (50–53) and found that gibel carp DICPs recruit SHP1 through the ITIM motif to inhibit the induction of IFN and interferon-stimulated gene (ISGs) (22). However, the molecular mechanism between SHP1 and IFN is still unknown. In this study, we first analyzed the diversification, evolution, and biased expression pattern of two *ptpn6* homeologs (*Cgptpn6-A* and *Cgptpn6-B*) in hexaploid gibel carp. Then, we explored the roles of *Cg*SHP1-A and *Cg*SHP1-B in the immune response regulation underlying their biased expression. Finally, we investigated the divergent mechanisms of *Cg*SHP1-A and *Cg*SHP1-B in regulating IFN through *in vitro* over-expression functional analysis.

## Materials and Methods

### Cells and Virus

*Epithelioma papulosum cyprini* (EPC) cells for western blotting and subcellular localization, Human embryonic kidney (HEK) 293T cells for coimmunoprecipitation (Co-IP) and dephosphorylation assays were cultured as described previously (54). Gibel carp brain (GiCB) cells for quantitative real-time PCR (qPCR) and viral infection were kindly provided by Prof. Zeng (Yangtze River Fisheries Research Institute, Chinese Academy of Fishery Sciences) (55). SVCV, a negative sense single-stranded RNA virus in the family Rhabdoviridae that could infect crucian carp and gibel carp (56) was propagated in GiCB cells until cytopathic effects (CPE) were observed, and then the culture media with cells were harvested and stored at -80°C until needed.

### Amplification of *Cg*SHP1 and Sequence Analysis

According to the genome sequences of gibel carp clone F, *Cgptpn6-A* and *Cgptpn6-B* cDNAs were amplified from gibel carp head kidney cDNA library by Rapid Amplification of cDNA Ends Polymerase Chain Reaction (RACE-PCR). PCR products amplified by a high-fidelity polymerase (TransGen Biotech) were purified and cloned into Trans5α Chemically Competent Cells. About 30 clones of each sample were sequenced and classified according to the specific SNPs among the sequences. The complete cDNA sequences of six *Cgptpn6* transcripts were deposited in GenBank (accession numbers from OK142786-OK142791). Amino acid sequences and domains were predicted by open reading frame (ORF) Finder (https://www.ncbi.nlm.nih.gov/orffinder/) and SMART (http://smart.embl-heidelberg.de/), multiple amino acid sequence alignment was performed by DNAman version 7.0 software. Phylogenetic tree was constructed by bootstrap analysis (1000 replicates) using the neighbor-joining method (NJ) in MEGA 7.0 software (57).

All the amino acid sequences used in this study were obtained from GenBank (http://www.ncbi.nlm.nih.gov/) and Ensembl (http://www.ensembl.org). The accession numbers are as following: *Homo sapiens* SHP1, NP_002822.2; *Mus musculus* SHP1, NP_038573.2; *Gallus gallus* SHP1, NP_001026655.1; *Lepisosteus oculatus* SHP1, ENSLOCT00000009309.1; *Danio rerio* SHP1, NP_956254.1; *Carassius auratus* SHP1-A, XP_026109501.1; *Carassius auratus* SHP1-B, XP_026139710.1. The exon-intron structure was determined by aligning cDNA and genomic sequences. Syntenic analyses were conducted by comparing the chromosomic regions around *ptpn6* genes in gibel carp chromosomes (*Cg*A16 and *Cg*B16) and crucian carp chromosomes (*Ca*A16 and *Ca*B16) with corresponding regions in *H. sapiens* chromosome 12, *M. musculus* chromosome 6, *G. gallus* chromosome 1, *L. oculatus* chromosome LG26, *D. rerio* chromosome 16. The genome information was obtained from the Ensembl genome database.

### Chromosome Preparation and Fluorescence *In Situ* Hybridization (FISH)

Chromosome preparation was performed as described previously (58). Five individuals of gibel carp clone F were injected phytohemagglutinin (PHA) (15-20 μg/g) *in vivo* and the head kidney cells were harvested by conventional hypotonic and fixation treatments. Briefly, the cells were exposed to a hypotonic solution for 30 min at room temperature and fixed for 30 min (with replacement of the fixative every 10 min without resuspension) in 3 ml of a 3:1 mixture of methanol and acetic acid. Finally, the cells were resuspended in 0.5 ml of fresh fixative and were spread on clean slides. The slides were prepared by the air-drying technique and storied at -20°C for FISH.

The bacterial artificial chromosome (BAC) clones containing *Cgptpn6-A* and *Cgptpn6-B* were screened by PCR. Then, *Cgptpn6-A*-BAC-DNA and *Cgptpn6-B*-BAC-DNA labeled by DIG-Nick Translation Mix and Biotin-Nick Translation Mix (Roche) respectively were used to perform FISH as described previously (35, 47). 4’, 6-diamidino-2-phenylindole (DAPI) was used to counterstain metaphase chromosomes. The results were acquired by Carl Zeiss upright fluorescence microscope Axio imager M2 (Analytical & Testing Center, IHB, CAS).

### RNA Extraction, Reverse Transcription, and Quantitative Real-Time PCR (qPCR)

Total RNAs from 12 adult tissues, including brain, kidney, intestine, skin, gill, heart, liver, muscle, spleen, thymus, ovary and head kidney, and GiCB cells were extracted by Trizol reagent (Invitrogen). RNase-free DNase was used to purify RNA by removing all contaminating genomic DNA. The first-strand cDNA was synthesized by using a GoScript Reverse Transcription System (Promega) according to the manufacturer’s instructions. qPCR was performed with Fast SYBR Green master mix (BioRad) on a CFX96 Real-Time System (BioRad). PCR conditions were as follows: 95°C for 5 min, then 40 cycles of 95°C for 20 s, 60°C for 20 s, 72°C for 20 s. *Eukaryotic translation elongation factor 1 alpha 1*, *like 1* (*eef1a1l1*) (M value = 0.74 < 1.5) was selected as the optimal reference gene for qPCR analysis according to the previous study (52). The primers of other IFN-related genes were also synthetized for qPCR (**Supplementary Table 1**). The specificity of the PCR amplification for all primer pairs was verified from the dissociation curves. The relative gene expression levels were calculated with 2^-△△CT^ method. All the samples were analyzed in triplicates.

### Plasmid Construction

For Coimmunoprecipitation assay (Co-IP) and Western blotting, the ORFs of *Cg*SHP1-A and *Cg*SHP1-B were cloned into pCMV-Myc, pCMV-HA (Clontech) and pcDNA3.1(+), respectively. For subcellular localization, the ORFs of *Cg*SHP1-A and *Cg*SHP1-B were inserted into pEGFP-N3 (Clontech) vector. Owing the extremely high amino acid sequence identities (98.46%-100.00%) of the IFN-related genes and autophagy-related genes between gibel carp and crucian carp (*C. auratus*) used in this study, we chose the corresponding plasmids from crucian carp constructed previously. The ORF of *C. auratus* mediator of IFN regulatory factor 3 (IRF3) activation (*Ca*MITA) [also called stimulator of interferon genes (STING)] (Gene accession number: MZ172421) and kinase TANK-binding kinase 1 (*Ca*TBK1) (Gene accession number: MZ172419) were inserted into pCS2-mCherry vector (Clontech Laboratories). The ORFs of mitochondrial antiviral signaling protein (*Ca*MAVS) (Gene accession number: MZ170793), *Ca*MITA, *Ca*TBK1, *Ca*IRF3 (Gene accession number: MZ172420), microtubule-associated Protein 1A/1B-Light Chain 3 (*Ca*LC3) (Gene accession number: XM_026238864.1), *Ca*Beclin1 (Gene accession number: XM_026249455.1), and autophagy-related gene 14 (*Ca*ATG14) (Gene accession number: XM_026286484.1) were cloned into pCMV-HA, pCMV-Myc and pCMV-Tag2c vector. Compared to the crucian carp genome, *Ca*MAVS, *Ca*MITA and *Ca*IRF3 localize in A subgenome, while *Ca*TBK1 and *Ca*ATG14 belong to B subgenome. The plasmids containing *Ca*IFN-luc and ISRE-Luc in pGL3-Basic luciferase reporter vectors were constructed as described previously (36). The primers including the restriction enzyme cutting sites used for plasmid construction were also listed in **Supplementary Table 1**. These primers were designed with Oligo Calc (Oligonucleotide Properties Calculator) (http://biotools.nubic.northwestern.edu/OligoCalc.html).

### Transient Transfection, Subcellular Localization and Virus Infection

Transient transfections were performed in EPC and GiCB cells seeded in 6-well or 24-well plates by using FishTrans Transfection Reagent (MeiSenTe Biotechnology) according to the manufacturer’s protocol [Total plasmid dosage (μg) and FishTrans (μl) dosage is at the ratio of 1:2]. For subcellular localization, EPC cells were plated onto coverslips in 6-well plates and transfected with indicated plasmids for 24 h. Following this, the cells were washed twice with phosphate-buffered saline (PBS) and fixed with 4% paraformaldehyde (PFA) for 1 h. After draining the fixative, the cells were stained with DAPI (1 μg/ml; Beyotime) for 5 min in a dark at room temperature. Finally, the coverslips were washed and observed with a Leica confocal microscope under a × 63 oil immersion objective (SP8; Leica Microsystems). Fluorescence intensity was analyzed with Image J.

For the antiviral assay, GiCB cells were seeded to 24-well plates and were transfected with 0.5 μg *Cg*SHP1-A and *Cg*SHP1-B or pcDNA3.1(+) vector, separately. At 24 h post-transfection, the GiCB cells were infected with SVCV at a multiplicity of infection (MOI = 0.01) and incubated at 28°C. At 48 h post-infection, the cell monolayers were washed with PBS, fixed with 4% PFA for 1 h, and stained with 0.05% crystal violet overnight to observe the CPE. The supernatants were subjected to 10-fold serial dilutions and then added (100 μl) onto a monolayer of GiCB cells cultured in a 96-well plate. After 48 h, the medium was removed and the cells were washed with PBS, fixed by 4% PFA and stained with 1% crystal violet. The virus titer was expressed as 50% tissue culture infective dose (TCID_50_/ml). Results are the representative of three independent experiments.

### Luciferase Activity Assay

EPC cells were seeded in 24-well plates, and 24 h later co-transfected with 250 ng luciferase reporter plasmid (*Ca*IFNpro-luc or ISRE-Luc), 250 ng *Cg*SHP1-A, *Cg*SHP1-B or pcDNA3.1(+), and 50 ng Renilla luciferase internal control vector (pRL-TK, Promega). Then, the cells were transfected again with a mimic of viral dsRNA poly I:C or the double-stranded DNA mimetic poly dA:dT at 24 h post-transfection, and the cells were infected by SVCV. To further explore the role of SHP1 in the RLR-induced interferon reaction, EPC cells were co-transfected with plasmids as described above, then transfected 250 ng *Ca*MAVS-, *Ca*MITA-, *Ca*TBK1- or *Ca*IRF3-Myc expressing plasmid or pCMV-Myc empty vector respectively. At 24 h post-transfection or infected, the cells were washed in PBS and lysed for measuring luciferase activity by Dual-Luciferase Reporter Assay System, according to the manufacturer’s instructions (Promega). Fireflyluciferase activities were normalized on the basis of Renilla luciferaseactivity. The results were the representative of more than three independent experiments, each performed in triplicate. Luciferase and qPCR assay data are expressed as the mean ± standard error of the mean (SEM). Error bars indicate the SEM (n = 3, biologically independent samples). Data were analyzed using a Student’s unpaired t-tests. A probability (*p*) < 0.05 was considered statistically significant (∗), and *p* < 0.01 was considered extremely significant (∗∗).

### *In Vitro* Protein Dephosphorylation Assay and Western Blotting

Transfected HEK 293T cells were lysed in radioimmuno-precipitation (RIPA) lysis buffer [1% NP-40, 50 mM Tris-HCl (pH 7.5), 150 mM NaCl, 1 mM EDTA, 1 mM NaF, 1 mM sodium orthovanadate,1 mM phenyl-methylsulfonyl fluoride, and 0.25% sodium deoxycholate] without phosphatase inhibitors. Protein dephosphorylation was carried out in 100 μl reaction mixtures consisting of 100 μg of cell protein and 10 U of calf intestinal phosphatase (CIP) (Sigma-Aldrich) (59). The reaction mixtures were incubated at 37°C for 1 h, then separated by 10% SDS-PAGE and transferred to polyvinylidene difluoride (PVDF) membrane (Bio-Rad). The membranes were blocked and incubated with indicated primary antibodies (Abs) at an appropriate dilution overnight at 4°C, washed three times with TBST buffer [25 mM Tris-HCl, 150 mM NaCl, 0.1% Tween 20 (pH 7.5)] and then incubated with secondary Abs. After additional three washes with TBST, the membranes were stained with Immobilon TM Western Chemiluminescent HRP Substrate (Millipore) and detected using an Image Quant LAS4000 system (GE Healthcare). Abs were diluted as follows: anti-β-actin (Cell Signaling Technology) at 1:3,000, anti-Flag (Sigma-Aldrich) at 1:3,000, anti-Myc (Santa Cruz Biotechnology) at 1:3,000, HRP-conjugated anti-mouse IgG or anti-rabbit IgG (Thermo Scientific) at 1:5,000. The results were the representative of three independent experiments.

### Coimmunoprecipitation Assay

HEK 293T cells seeded in 10 cm^2^ dishes overnight were transfected with a total of 10 µg of the plasmids. At 24 h post-transfection, medium was removed carefully and cell monolayer was washed twice with 10 ml ice-cold PBS. Then the cells were lysed in 1 ml of RIPA lysis buffer containing protease inhibitor mixture (Sigma-Aldrich) at 4°C for 1 h on a rocker platform. The cellular debris was removed by centrifugation at 12,000 × g for 15 min at 4°C. The 100 μl supernatant was transferred to a fresh tube and the rest was incubated with 30 μl of anti-Flag or anti-Myc affinity gel (Sigma-Aldrich) overnight at 4°C with constant agitation. Immunoprecipitated proteins were collected by centrifugation at 5,000 × g for 1 min at 4°C, washed three times with lysis buffer, and resuspended in 100 μl SDS sample buffer (59). The immunoprecipitates and whole cell lysates were analyzed by western blotting with the indicated Abs.

## Results

### Two Divergent *Cgptpn6* Homeologs With Conserved Genomic Structure in Gibel Carp

Six *Cgptpn6* transcripts cloned from gibel carp head kidney were clearly clustered into two homeologs (*Cgptpn6-A* and *Cgptpn6-B*), and each of them clearly possessed three alleles (**Supplementary Figure 1**). The average identities among the three alleles (99.87% ± 0.09% for *Cgptpn6-A* and 99.73% ± 0.05% for *Cgptpn6-B*) were higher than that between *Cgptpn6-As* and *Cgptpn6-Bs* (89.28% ± 0.07%). The major differences between the *Cgptpn6-As* and *Cgptpn6-Bs* homeologs were observed in the 3′ untranslated region (UTR), where the sequence identity was only 73.10%. The ORFs of the three *Cgptpn6-A* alleles were all 1761 bp, encoding two *Cg*SHP1-A proteins [*Cg*SHP1-A1 and *Cg*SHP1-A2/A3, 586 amino acids (aa)] with one aa difference at the 29th. The ORFs of the three *Cgptpn6-B* genes were also 1761 bp, encoding the same *Cg*SHP1-B protein (586 aa, *Cg*SHP1-B1/B2/B3). All gibel carp SHP1 proteins possessed three conserved domains (two SH2-domains [N-SH2, C-SH2] and a PTPc domain). The PTP signature motif (I/VHCSAGIGRTG) is identical among mammals and fish SHP1 (**Supplementary Figure 2A**). Multiple sequence alignments and phylogenetic analysis showed that almost identical (99.08% and 100.00%) gibel carp and crucian carp SHP1-A and SHP1-B were grouped into two separate branches, implying the duplication of the *ptpn6* gene in the common ancestor of gibel carp and crucian carp, which then clustered with zebrafish SHP1 and spotted gar SHP1 (**Supplementary Figure 2B**).

**Figure 1:**
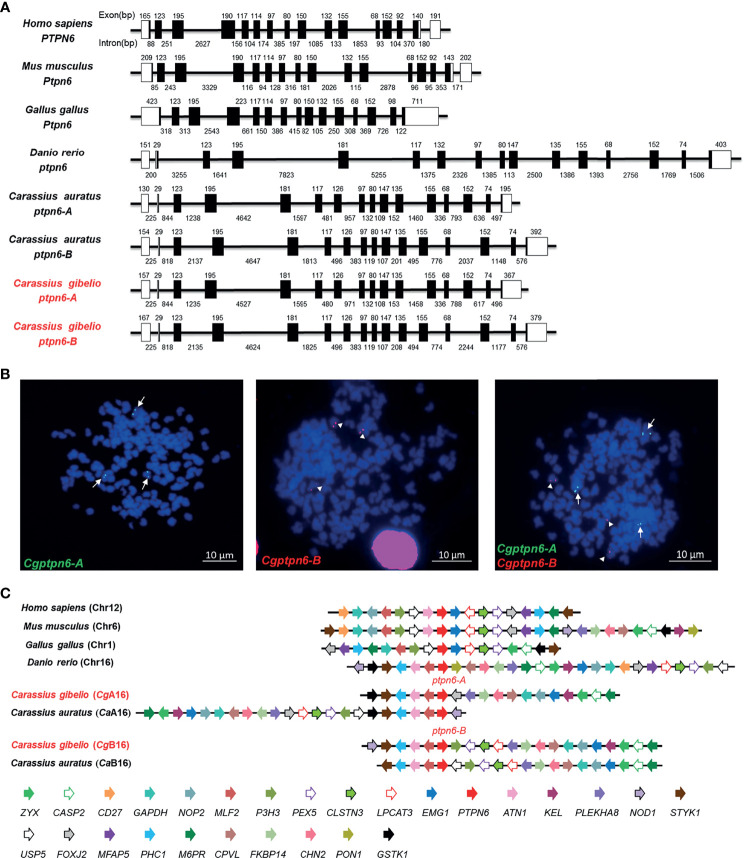
Molecular characterization of *Cgptpn6-A* and *Cgptpn6-B* in gibel carp. **(A)** Genomic structure of *ptpn6* genes. Exons and introns are shown by boxes and horizontal lines, respectively. ORFs are highlighted by black boxes. The exon and intron size are indicated upon or below themselves as base pairs (bp). **(B)** Localization of *Cgptpn6-A* (green, indicated by arrows) and *Cgptpn6-B* (red, indicated by arrowheads) on metaphase chromosomes (blue). Scale bars = 10 μm **(C)** Syntenic alignment of chromosomal regions around vertebrate *ptpn6* genes. *Cgptpn6-A* and *Cgptpn6-B* are located on the chromosome *Cg*A16 and *Cg*B16 respectively. Chromosome segments are represented as thick lines. The conserved gene blocks are shown in matching colors and the transcription orientation are indicated by arrows.

Subsequently, the genomic structure and syntenic alignment of gibel carp *ptpn6-A* and *ptpn6-B* and other vertebrates were identified. Except chicken *Ptpn6* (15 exons), both *Cgptpn6-A* and *Cgptpn6-B*, as well as other vertebrate *ptpn6*, were composed of 16 exons (**Figure 1A**). *Cgptpn6-A* and *Cgptpn6-B* possess almost identical lengths of exons except the first and last exons, which are the loci for transcribing the 5′ and 3′ UTR. However, the lengths of their introns are varied and their identity is only 52.70%. The identities between the corresponding introns of *Cgptpn6-A* and *Cgptpn6-B* ranged from 19.82% (11th intron) to 90.74% (9th intron). Similarities in the genomic structure between crucian carp *ptpn6-A* and *ptpn6-B* were also observed. In addition, only the lengths of the 1st, 7th, and 16th exons of *Cgptpn6-A* and *Cgptpn6-B* are different from zebrafish *ptpn6*, implying a highly conserved genomic structure in Cyprinidae fish. Human *PTPN6* and mouse *Ptpn6* have similar exon lengths, which are different from chicken and fish *ptpn6* genes.

Each *Cgptpn6* homeolog with three alleles was confirmed by FISH. Consistent with our previous studies (35, 36), three green *Cgptpn6-A* signals and three red *Cgptpn6-B* signals were located on the three different chromosomes when simultaneously using *Cgptpn6-A*-BAC-DNA and *Cgptpn6-B*-BAC-DNA as probes respectively (**Figure 1B**). Syntenic alignment showed that gibel carp chromosome *Cg*A16 and *Cg*B16 both retained approximately 60% of the analyzed homologous genes in zebrafish chromosome 16 and had a conserved gene block (*styk1-phc1-atn1-mlf2-ptpn6*). One homeolog of the other duplicated genes in *Cg*A16 and *Cg*B16 seemed to be deleted, and became singletons. For example, *p3h3*, *pex5*, *clstns*, *lpcat3*, and *nod1* were mapped only in *Cg*B16, while *foxj2* and *gstk1* ware located only in *Cg*A16 (**Figure 1C**).

### Dominant Expression of *Cgptpn6-A* in Gibel Carp Adult Tissues

**Figure 2:**
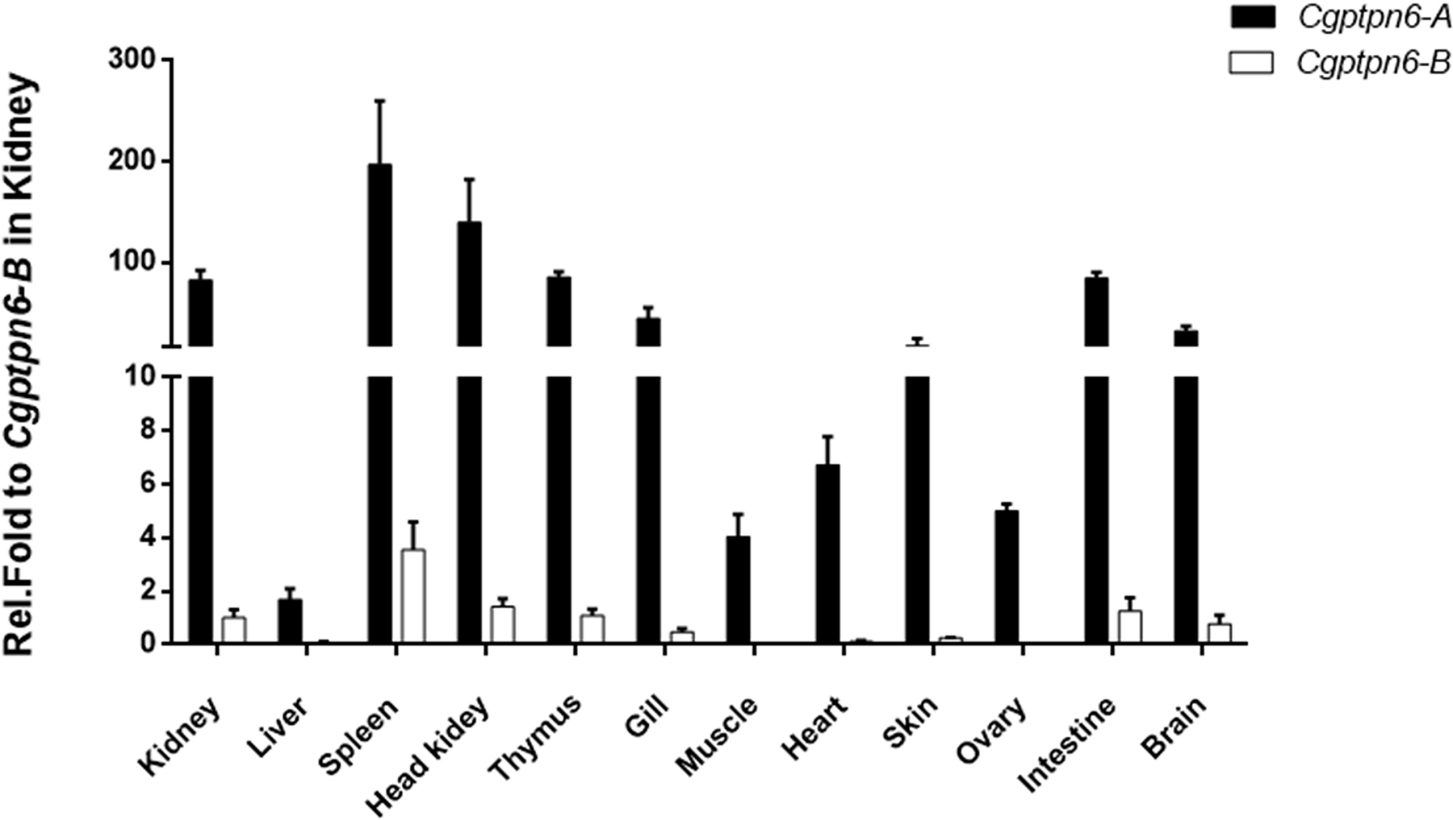
qPCR analysis of *Cgptpn6-A* and *Cgptpn6-B* expression in healthy adult tissues. *eef1a1l1* was used as control. Each bar represents mean ± standard deviation (SD) (n = 3).

The distributions of *Cgptpn6-A* and *Cgptpn6-B* in 12 adult tissues of gibel carp were analyzed by qPCR. Two specific pairs of primers were designed to amplify *Cgptpn6-A* and *Cgptpn6-B*, respectively. *Cgptpn6-A* and *Cgptpn6-B* were abundantly expressed in the immune-related tissues, such as spleen, head kidney, kidney, and thymus (**Figure 2**). *Cgptpn6-A* expression was remarkably higher (20-1726 folds) than that of *Cgptpn6-B* in all tissues, suggesting that *Cgptpn6-A* may play a dominant role in immune regulation.

### Higher Upregulated Expression of *Cgptpn6-A* Induced by Poly I:C, Poly dA:dT, and SVCV

**Figure 3:**
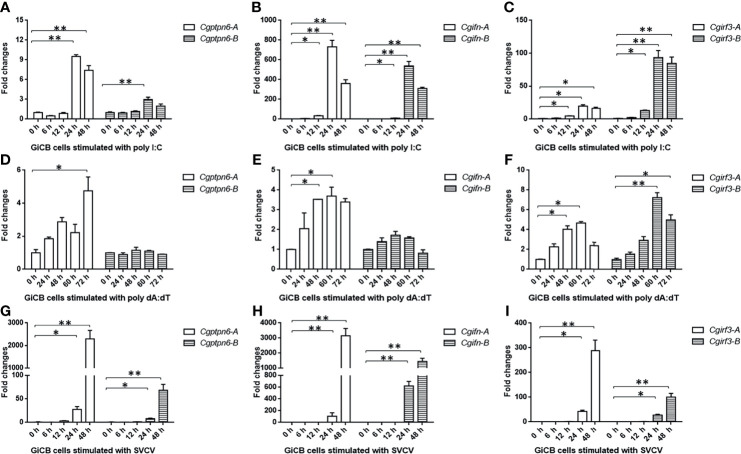
Dynamic expression changes of *Cgptpn6-A* and *Cgptpn6-B*, *Cgifn-A* and *Cgifn-B*, *Cgirf3-A* and *Cgirf3-B* stimulated by 1 μg poly I:C **(A–C)**, poly dA:dT **(D–F)** or SVCV (MOI = 1) in GiCB cells **(G–I)** by qPCR analyses. *eef1a1l1* was used as an internal control for normalization and the relative expression is represented as fold induction relative to the expression level in control cells. Each bar represents mean ± standard deviation (SD) (n = 3). Asterisks indicate significant differences from control (*p < 0.05, **p < 0.01).

Subsequently, the dynamic expression changes of *Cgptpn6-A* and *Cgptpn6-B* were investigated after stimulation with poly I:C, poly dA:dT, and SVCV. *Cgptpn6-A* and *Cgptpn6-B* expression increased up to 9.5-fold and 3-fold, respectively, at 24 h after poly I:C treatment (**Figure 3A**). Poly dA:dT showed a weaker stimulation (4.7-fold at 72 h) for the upregulation of *Cgptpn6-A* expression and showed no effect on the expression of *Cgptpn6-B* (**Figure 3D**). In contrast, *Cgptpn6-A* and *Cgptpn6-B* expression were remarkably up-regulated (2293- and 68-fold respectively) at 48 h after SVCV infection (**Figure 3G**). These results indicate that *Cgptpn6-A* and *Cgptpn6-B* may both participate in the host immune response, with *Cgptpn6-A* playing a dominant role. Similar to *Cgptpn6*, the other IFN-related genes, such as *Cgifn*, *Cgirf3*, *Cgrig-i*, and *Cgviperin*, all have two homeologs (52). *Cgifn-A* and *Cgifn-B*, as well as *Cgirf3-A* and *Cgirf3-B*, showed similar dynamic expression changes (**Figures 3B, C, E, F, H, I**) as *Cgptpn6-A* and *Cgptpn6-B*, implying potential association between *Cgptpn6* and IFN response.

### *Cg*SHP1-A and *Cg*SHP1-B Both Negatively Regulate IFN Response

**Figure 4:**
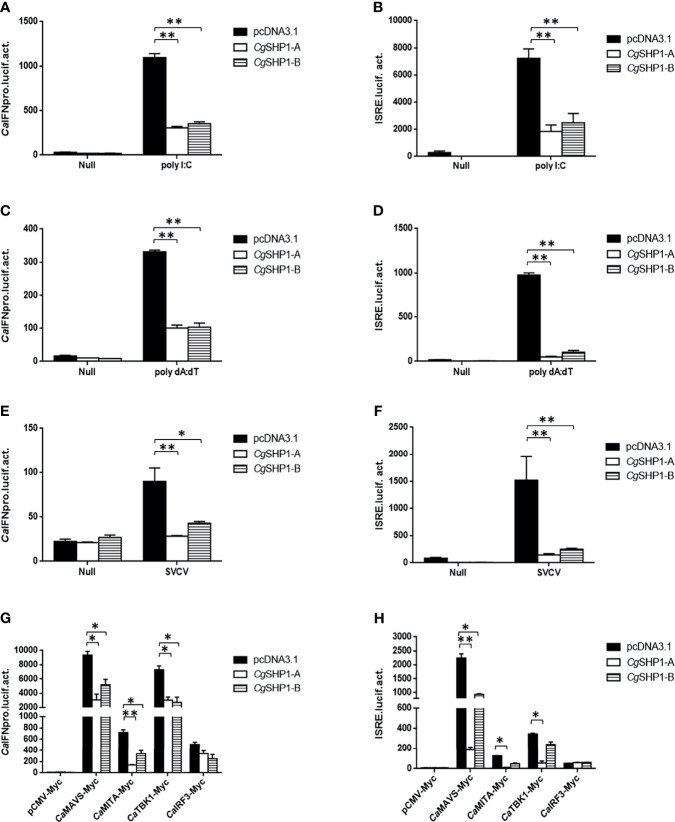
*Cg*SHP1-A and *Cg*SHP1-B inhibit IFN response induced by 1 μg poly I:C **(A, B)**, poly dA:dT **(C, D)**, SVCV (MOI = 1) **(E, F)** in EPC cells. *Cg*SHP1-A and *Cg*SHP1-B inhibit *Ca*MAVS, *Ca*MITA and *Ca*TBK1-mediated activation of *Ca*IFNpro and ISRE **(G, H)**. Data are expressed as mean ± SEM, n = 3. Asterisks indicate significant differences from control (*p < 0.05, **p < 0.01).

To explore the association between *Cgptpn6* and innate immunity, the effects of *Cgptpn6-A* and *Cgptpn6-B* on IFN regulation were examined. The overexpression of *Cg*SHP1-A and *Cg*SHP1-B both remarkably inhibited *Ca*IFN promoters and ISRE activities induced by poly I:C, poly dA:dT, and SVCV (**Figures 4A–C**). In comparison with *Cg*SHP1-B, only a slightly stronger inhibition of *Cg*SHP1-A was observed. Previous studies showed that fish IFN response could be triggered through the RLR signaling pathway (60). As shown in **Figure 4D**, the co-transfection of *Cg*SHP1-A or *Cg*SHP1-B can obviously inhibit the activities of *Ca*IFNpro and ISRE promoted by *Ca*MAVS, *Ca*MITA, and *Ca*TBK1. The repression effects seemed to be stronger by the co-transfection of *Cg*SHP1-A relative to *Cg*SHP1-B. In addition, both *Cg*SHP1-A and *Cg*SHP1-B had no significant effect on the activities of *Ca*IFNpro and ISRE induced by *Ca*IRF3.

**Figure 5:**
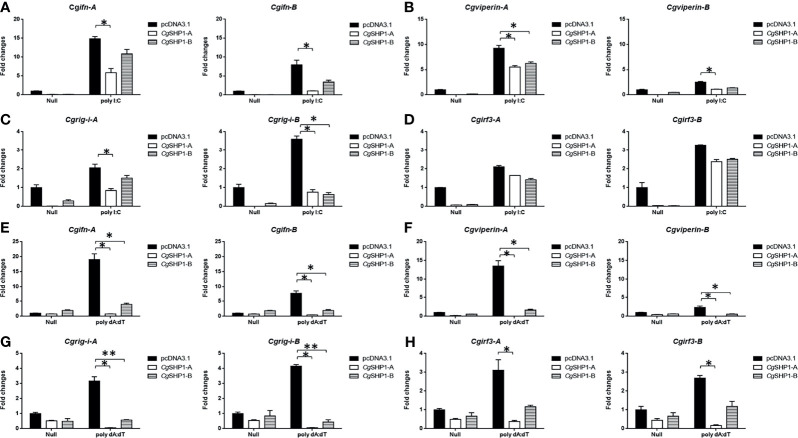
*Cg*SHP1-A and *Cg*SHP1-B inhibit the expression of gibel carp IFN and IFN-related genes induced by 1 μg/ml poly I:C **(A–D)** or poly dA:dT **(E–H)** in GiCB cells. *eef1a1l1* was used as control and the relative expression is represented as fold induction relative to the expression level in control cells (set to 1). Each bar represents mean ± SEM (n = 3). The asterisks indicate the significant differences (*p < 0.05, **p < 0.01).

These results were further supported by qPCR findings. The upregulated expression of RLR molecules (*Cgrig-i-A* and *Cgrig-i-B*), *ifn* (*Cgifn-A* and *Cgifn-B*), and ISGs (*Cgviperin-A* and *Cgviperin-B*) induced by poly I:C or poly dA:dT were remarkably reduced by *Cg*SHP1-A or *Cg*SHP1-B overexpression. Similarly, the inhibitory effect of *Cg*SHP1-A was more significant than that of *Cg*SHP1-B (**Figures 5A, B, E, F**). Similar to the results of the luciferase activity assay, the increased expression of *Cgirf3-A* and *Cgirf3-B* were rarely influenced by the *Cg*SHP1-A or *Cg*SHP1-B overexpression induced by poly I:C (**Figures 5C, D**), while the upregulated expression of *Cgirf3-A* and *Cgirf3-B* were decreased by the *Cg*SHP1-A or *Cg*SHP1-B overexpression induced by poly dA:dT (**Figures 5G, H**). These data demonstrate that *Cg*SHP1-A and *Cg*SHP1-B may negatively regulate IFN response through the RLR signaling pathway.

### *Cg*SHP1-A and *Cg*SHP1-B Are Both Associated With *Ca*TBK1 and Inhibit *Ca*TBK1-Induced Phosphorylation of *Ca*MITA

**Figure 6:**
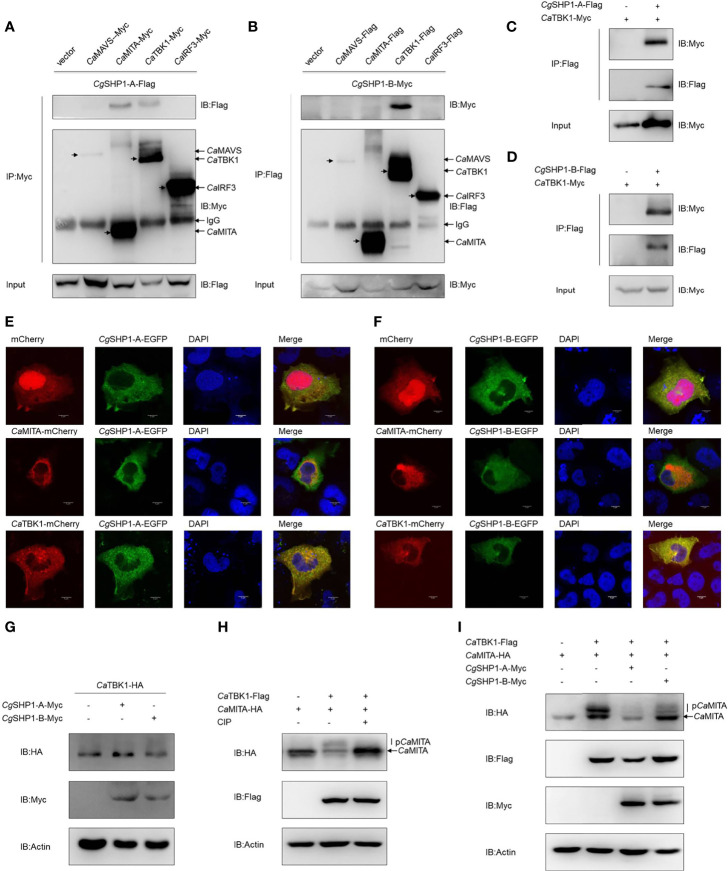
Interaction of *Cg*SHP1s with *Ca*TBK1 and *Ca*MITA. **(A–D)** Co-IP analyses between *Cg*SHP1s and *Ca*TBK1, *Ca*MITA in HEK 293T cells. **(E, F)** Subcellular localization of *Cg*SHP1-A and *Cg*SHP1-B with *Ca*TBK1 and *Ca*MITA in EPC cells. Scale bars = 5 μm. **(G)** *Cg*SHP1-A and *Cg*SHP1-B do not influence the expression of *Ca*TBK1 in EPC cells. **(H, I)** Co-IP analyses reveal *Ca*TBK1-mediated phosphorylation of *Ca*MITA was inhibited by CIP (10 U) in HEK 293T cells **(H)**, *Cg*SHP1-A and *Cg*SHP1-B decrease the phosphorylation of *Ca*MITA induced by *Ca*TBK1 **(I)**. All experiments were repeated for at least three times with similar results.

To further decipher the relationship between *Cgptpn6* and the RLR signaling pathway, Co-IP experiments were performed using *Cg*SHP1-A-Flag and RLR cascades with the Myc tag (*Ca*MAVS-Myc, *Ca*MITA-Myc, *Ca*TBK1-Myc and *Ca*IRF3-Myc). The results clearly showed that *Cg*SHP1-A was efficiently associated with *Ca*MITA and *Ca*TBK1, not with *Ca*MAVS and *Ca*IRF3 (**Figure 6A**). However, *Cg*SHP1-B-Myc could only be efficiently pulled down by *Ca*TBK1-Flag (**Figure 6B**). The interactions between *Cg*SHP1-A and *Cg*SHP1-B with *Ca*TBK1 were confirmed by the reverse assays (**Figures 6C, D**). The subcellular locations of *Cg*SHP1s and *Ca*MITA or *Ca*TBK1 were also monitored in EPC cells. Consistent with the findings of a previous report (61), *Cg*SHP1-A and *Cg*SHP1-B both localized in the cytosol, implying that SHP1 is an intracellular cytoplasmic signaling enzyme (**Figures 6E, F**). The colocalization results showed that the green fluorescence signals of *Cg*SHP1-A or *Cg*SHP1-B were uniformly overlapped with the red signals of *Ca*TBK1, and partly overlapped with the *Ca*MITA signals in the cytosol (**Figures 6E, F**).

Next, we investigated the protein changes to determine the effect of *Cg*SHP1-A and *Cg*SHP1-B on the *Ca*TBK1. Overexpression of *Cg*SHP1-A or *Cg*SHP1-B exerted little influence on the expression of *Ca*TBK1 (**Figure 6G**). Since SHP1 is known as protein tyrosine phosphatase, we speculated that *Cg*SHP1-A and *Cg*SHP1-B may affect the post-translational status of some downstream molecules phosphorylated by TBK1. We first confirmed that *Ca*MITA was indeed phosphorylated by *Ca*TBK1. When *Ca*MITA was co-transfected with *Ca*TBK1, weakly shifted bands with higher molecular weights were detected. As expected, these bands disappeared after treatment with CIP (**Figure 6H**). We subsequently investigated the role of *Cg*SHP1-A and *Cg*SHP1-B in *Ca*TBK1 kinase activity. The phosphorylated *Ca*MITA was reduced with overexpression of *Cg*SHP1-A or *Cg*SHP1-B. Interestingly, *Cg*SHP1-A degrades unphosphorylated *Ca*MITA (**Figure 6I**). Taken together, these data demonstrate that both *Cg*SHP1-A and *Cg*SHP1-B inhibit *Ca*TBK1-induced phosphorylation of *Ca*MITA.

### *Cg*SHP1-A Degrades *Ca*MITA *via* an Autophagy Pathway

**Figure 7:**
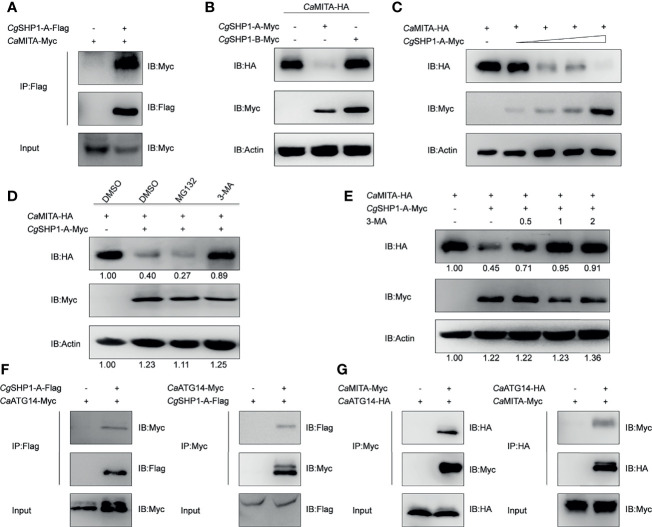
*Cg*SHP1-A interacts with and degrades *Ca*MITA by autophagy pathway. **(A)** Co-IP analysis between *Cg*SHP1-A and *Ca*MITA. **(B–D)** *Cg*SHP1-A degrades *Ca*MITA in a dose-dependent manner by autophagy pathway. 1.5 μg *Ca*MITA-HA co-transfected with 1.5 µg *Cg*SHP1s-Myc **(B)** and various concentration of *Cg*SHP1-A-Myc (0.5 μg, or 1 μg, or 1.5 μg or 2 μg, empty vector was used to make up the rest) **(C)** in EPC cells. At 18 h post-transfection, the cells were treated with DMSO, MG132 and 3-MA for 6 h **(D)**. The cell lysates were subjected to IB. Experiments were repeated for at least three times with similar results**. (E)** Effects of 3-MA on *Cg*SHP1-A mediated destabilization of *Ca*MITA. Transfection with the indicated expression vectors (2 μg/well) and treated with DMSO or 3-MA (0.5, 1, or 2 mM) for 6 h at 18 h post-transfection, the WCLs were analyzed by IB. **(F, G)** Co-IP of *Cg*SHP1-A-Flag with *Ca*ATG14-Myc **(F)** or *Ca*MITA-Myc with *Ca*ATG14-HA **(G)** in HEK 293T cells.

The interaction between *Cg*SHP1-A and *Ca*MITA was further confirmed by the reverse assay (**Figure 7A**). To determine the effect of *Cg*SHP1-A on *Ca*MITA, *Cg*SHP1-A was cotransfected with *Ca*MITA. Overexpression of *Cg*SHP1-A caused a significant reduction of *Ca*MITA in a dose-dependent manner (**Figures 7B, C**). Consistent with the no or very weak interaction between *Cg*SHP1-B and *Ca*MITA (**Figure 6B**), the overexpression of *Cg*SHP1-B did not reduce *Ca*MITA level (**Figure 7B**). A proteasome inhibitor (MG132) and an autophagy–lysosomal pathway inhibitor 3-methyladenine (3-MA) were used to examine the process underlying the *Cg*SHP1-A-mediated *Ca*MITA degradation. In comparison with the control (DMSO treatment) and MG132 groups, 3-MA could effectively block the degradation of *Ca*MITA induced by *Cg*SHP1-A in a dose-dependent manner (**Figures 7D, E**), implying that *Cg*SHP1-A can degrade *Ca*MITA *via* an autophagy-lysosomal pathway. To test this speculation, we preliminarily evaluated several autophagic components to identify which one could interact with *Cg*SHP1-A. Co-IP assays showed that *Cg*SHP1-A interacted with *Ca*ATG14 (**Figure 7F**). Similarly, the interaction between *Ca*MITA and *Ca*ATG14 was also confirmed (**Figure 7G**). These data demonstrate that *Cg*SHP1-A can degrade *Ca*MITA probably through *Ca*ATG14-mediated autophagy signaling pathway.

### Both *Cg*SHP1-A and *Cg*SHP1-B Attenuate the Cellular Antiviral Response

**Figure 8:**
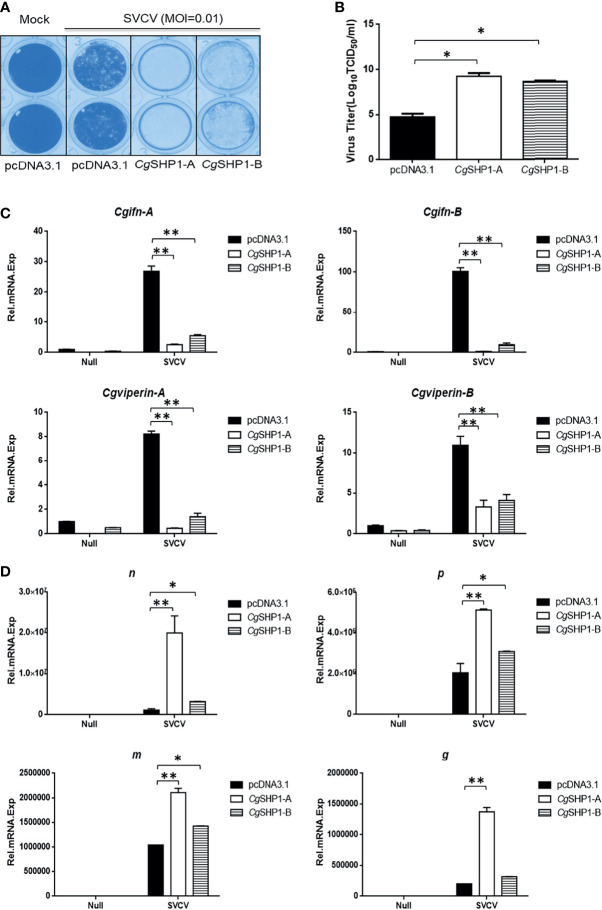
*Cg*SHP1-A and *Cg*SHP1-B attenuate the cellular antiviral response. **(A, B)** Enhance of virus replication by overexpression of *Cg*SHP1-A or *Cg*SHP1-B. GiCB cells were transfected with 0.5 μg pcDNA3.1-*Cg*SHP1-A or *Cg*SHP1-B or empty vector. At 24 h post-transfection, cells were infected with SVCV (MOI = 0.01) for 48 h **(A)**. Viral titer was measured according to the method of Karber **(B)**. **(C)** *Cg*SHP1-A and *Cg*SHP1-B inhibit the expression of *Cgifn-A* and *Cgifn-B*, *Cgviperin-A* and *Cgviperin-B*. GiCB cells were transfected with 2 μg pcDNA3.1-*Cg*SHP1-A or *Cg*SHP1-B or empty vector. At 24 h post-transfection, cells were untreated or infected with SVCV (MOI = 1). After 24 h infection, the WCLs were detected for qPCR analysis. **(D)** The mRNA levels of cellular *n*, *p*, *m* and *g.* The same samples were prepared similarly as described above for **(C)**. The relative transcriptional levels were normalized to the transcriptional level of the *eef1a1l1* gene and were represented as fold induction relative to the transcriptional level in the control cells. Data are expressed as mean ± SEM, n = 3. Asterisks indicate significant differences from control values (* *p* < 0.05, **p < 0.01).

Since *Cg*SHP1-A and *Cg*SHP1-B negatively regulate the IFN response, the modulation of *Cg*SHP1 to the antiviral innate immune response was evaluated. In comparison with empty vector control infected with SVCV (MOI = 0.01), the overexpression of *Cg*SHP1-A or *Cg*SHP1-B in GiCB cells both resulted in an enhanced CPE (**Figure 8A**) and the viral titers increased about 10^4.5^- and 10^3.94^-fold respectively at 2 days post-infection (**Figure 8B**). In addition, the upregulated expression of *Cgifn-A*, *Cgifn-B*, *Cgviperin-A* and *Cgviperin-B* induced by SVCV were remarkably repressed by overexpression of *Cg*SHP1-A or *Cg*SHP1-B (**Figure 8C**). The inhibition effects of *Cg*SHP1-A transfection were stronger than those of *Cg*SHP1-B transfection. Meanwhile, more abundant transcripts of SVCV genes, *n*, *p*, *m* and *g* were detected in the *Cg*SHP1-A overexpressed group (**Figure 8D**). These data indicate that both *Cg*SHP1-A and *Cg*SHP1-B negatively regulate the cellular antiviral response, in which *Cg*SHP1-A is potentially dominant.

## Discussion

Protein tyrosine phosphorylation, an important post-translational modification, is necessary for normal immune regulation and occurs under the strict control of PTKs-PTPs (1, 62, 63). Because of the variable activities and poor substrate specificity of PTPs, research on these molecules has seriously lagged behind PTKs, and they have only recently begun to attract considerable attention as potential therapeutic targets (4). One of our previous studies on gibel carp disease resistance breeding showed that SHP1 can be recruited to inhibitory immune receptor DICPs, which could inhibit the expression of IFN and ISGs (22). In this study, we first identified two diverged gibel carp *ptpn6* homeologs and observed the dominant expression of *Cgptpn6-A*. Then, we revealed that *Cg*SHP1-A and *Cg*SHP1-B both negatively regulate the IFN response through the RLR signaling pathway. Finally, we identified the dominant role of *Cg*SHP1-A in negatively regulating cellular antiviral response.

One of the most interesting consequences of hybridization and polyploidization is the diversification of duplicated genes. Allopolyploids, which arise from interspecific hybridization, possess duplicated gene copies (64). Because gibel carp is derived from a common allotetraploid ancestor by autotriploidy about 0.8 million years ago, it possesses the same haplotype (A+B) as curcian carp. It means that gibel carp and crucian carp generally have the very high similar genes but the former has one more allele. Consistently, gibel carp also showed two *ptpn6* homeologs with about 90% identity, and each of homeologs possesses three alleles with identities above 99% (**Supplementary Figure 1**). Together with other conserved genes (i.e., *dmrt1*, *foxl2*, *viperin*, *nanos2* and *bmp15*), the phylogeny of *Cgptpn6* confirmed the assumption that gibel carp and crucian carp are derived from a common allotetraploid ancestor, and a subsequent autotriploidy event drove the speciation of gibel carp.

Under relaxed purifying selection, duplicated homeologs may step into different evolutionary trajectories: co-retained or fractionated (one of the duplicated genes is either retained or deleted) (33). Approximately 60% of the analyzed homologous genes in zebrafish chromosome 16 were co-retained both in gibel carp chromosome *Cg*A16 and *Cg*B16, while the others had fractionated. In addition, *pon1*, *cd27*, and *mfap5* were also located neither in *Ca*A16 nor in *Ca*B16, implying that they might have been lost in the ancestor of *Carassius* complex after an allotetraploidy event. Relative to *Ca*A16, a gene block (*lpcat3*-*clstns-pex5*-*p3h3*) is not in *Cg*A16 (**Figure 1C**), which suggests that it might have been deleted in gibel carp after divergence from crucian carp. Similar to the results observed in zebrafish and Nile tilapia (23, 24), *Cgptpn6s* is ubiquitously expressed in the analyzed tissues and abundantly in immune tissues (i.e., spleen, head kidney, and thymus) (**Figure 2**). Numerous examples have proven that homeolog biased expression seems to be a rule rather than an exception (30). In our previous studies, homeolog bias occurred differently in different tissues (35, 36, 50); for example, *Cgviperin-A* was expressed higher than *Cgviperin-B* in the spleen and liver, whereas in the gill, the transcripts of *Cgviperin-B* were more abundant than *Cgviperin-A* (36). However, *Cgptpn6-A* was dominant in the 12 adult tissues (**Figure 2**), and poly I:C, poly dA:dT, and SVCV all induced higher upregulated expression of *Cgptpn6-A* relative to *Cgptpn6-B* (**Figure 3**). The expression dominance of *Cgptpn6-A* implies its leading role in immune regulation.

As the first defense line, the innate immune system, including IFNs, plays vital roles against invasive pathogens (65, 66) and is tightly regulated by complex mechanisms that prevent excessive inflammation and autoimmunity (67–69). SHP1 is known to be a major regulator in this process (70, 71). For example, the inducible deletion *of Ptpn6* led to an increase in IFNγ expression in the *Ptpn6* ^fl/fl^ERT2*-*Cre mouse (72). Epstein-Barr virus (EBV) Tegument protein BGLF2 facilitates the recruitment of SHP1 to STAT1, which reduces STAT1 phosphorylation and thereby the induction of IFN and ISGs in HEK293 cells (73). The overexpression of mouse SHP-1 in L929 cells markedly reduced the phosphorylation of several critical signaling regulators (i.e., TBK1, IRF3, STAT1, p65, p38, and Erk) and thereby inhibited type I IFN production in response to vesicular stomatitis virus infection (74). However, IFN-β induced by poly (I:C) was significantly impaired in the splenocytes of SHP-1-deficient mouse both *in vitro* and *in vivo* (75). In this study, the overexpression of *Cg*SHP1-A or *Cg*SHP1-B both inhibited the IFN response stimulated by poly I:C, poly dA:dT, and SVCV (**Figure 4**), indicating that fish SHP1 is also a critical negative factor for IFN. The upregulated expression of *Cgirf3s* were more significantly decreased by the *Cg*SHP1-A or *Cg*SHP1-B overexpression induced by poly dA:dT than poly I:C (**Figure 5**). Poly dA:dT has been reported to trigger not only the RIG-I pathway but also the cGAS-STING/MITA pathway to induce type I IFN (76). Besides, MITA as the target of *Cg*SHP1-A participates in both cytoplasmic RNA- and DNA-triggered signaling pathways that converge on the TBK1-IRF3 axis in different molecular mechanisms (77). Therefore, we speculate that overexpression of *Cg*SHP1-A or *Cg*SHP1-B could more significantly reduce the increased expression of *Cgirf3s* triggered by poly dA:dT through two pathways. Moreover, overexpression of *Cg*SHP1-A or *Cg*SHP1-B both promoted SVCV proliferation, and the overexpression of *Cg*SHP1-A had a more powerful effect on the suppression of IFN response than that of *Cg*SHP1-B, while CPE in the GiCB transfected with *Cg*SHP1-A was more obvious than that of *Cg*SHP1-B (**Figure 8**). These results indicate that *Cg*SHP1-A and *Cg*SHP1-B negatively regulate gibel carp antiviral activities, in which the former plays a dominant role.

The importance of SHP1 has been implicated in various signaling events in mammals, including adaptive immunity pathways such as the T cell receptor (TCR) and BCR signaling pathway (78, 79), and innate immunity pathways, including Janus kinase-signal transducer and activator of transcription (JAK-STAT), phosphatidylinositol 3-kinase (PI3-K)/activation of protein kinase B (Akt), mitogen-activated protein kinases (MAPKs), and transcription nuclear factor (NF-κB) pathways, and Toll-like receptor (TLR) signaling pathway (13, 15, 73, 75, 80–82). For example, SHP-1 inhibited the TLR-mediated proinflammatory cytokine production by repressing the activation of MAPKs and NF-κB, but it increased TLR- and RIG-I-activated IFN-β production by inhibiting IRAK1 activation in mouse splenocytes after VSV infection (75). In this study, we found that both *Cg*SHP1-A and *Cg*SHP1-B were associated with *Ca*TBK1 and could inhibit *Ca*TBK1-induced phosphorylation of *Ca*MITA, and *Cg*SHP1-A degrades unphosphorylated *Ca*MITA (**Figure 6**). The activation of the fish IFN response has been well-characterized (65, 83, 84). Similar to the process in mammals, viral products are recognized by TLRs or RLRs and then trigger an IRF3/7-dependent IFN response. In the RLR-activated IFN signaling cascade, members of the RLR family, such as RIG-I, interacts with MAVS that subsequently associates with TBK1 and MITA, which enables the phosphorylation of IRF3/7 for translocating into the nucleus and then triggering the production of IFNβ (60, 65). TBK1 and MITA are strictly regulated to achieve a coordinated response, and several negative regulatory molecules for these factors have been identified. For example, zebrafish major vault protein (MVP) inhibits IFN production through recruitment and degradation of TBK1 in a lysosome-dependent manner (85), and transmembrane protein 33 (TMEM33) acts as a competitive substrate of TBK1 to reduce MITA/IRF3 phosphorylation (86). According to previous report, the C-terminal domain of SHP2, which has a similar structure to SHP1, directly bounds TBK1 by interacting with the kinase domain of TBK1 (87). Therefore, we speculate that *Cg*SHP1-A and *Cg*SHP1-B might also interact with *Ca*TBK1 *via* the kinase domain of *Ca*TBK1 and inhibit the kinase domain of *Ca*TBK1-induced phosphorylation of *Ca*MITA. Since TBK1 is a serine/threonine-kinase and its induced phosphorylation is not tyrosine phosphorylation (88, 89), the inhibition of *Ca*TBK1-induced phosphorylation by *Cg*SHP1s may be independent of its tyrosine phosphatase activity, which requires further investigation. Interestingly, we observed that only *Cg*SHP1-A could degrade *Ca*MITA *via* an autophagy pathway (**Figure 7**). Autophagy is one of major cellular protein degradation pathways to decompose misfolded/unfolded proteins or invading cytoplasmic organisms in eukaryotes (90, 91). Many recent studies have reported that autophagy has a negative influence on type I IFN signaling pathways (92–95). Taken together, the differential expression pattern and regulative mechanisms indicate that *Cg*SHP1-A and *Cg*SHP1-B might have sub-functionalized and that *Cg*SHP1-A overwhelmingly dominates *Cg*SHP1-B. Besides the coding sequences, mutations in cis-elements may have also led to the subfunctionalization of duplicated genes (33, 96). Further research will be required to identify the distinct motifs or sites between *Cgptpn6-A* and *Cgptpn6-B*, including coding sequences and promoters, which will result in their differential expression and regulative mechanisms.

**Figure 9:**
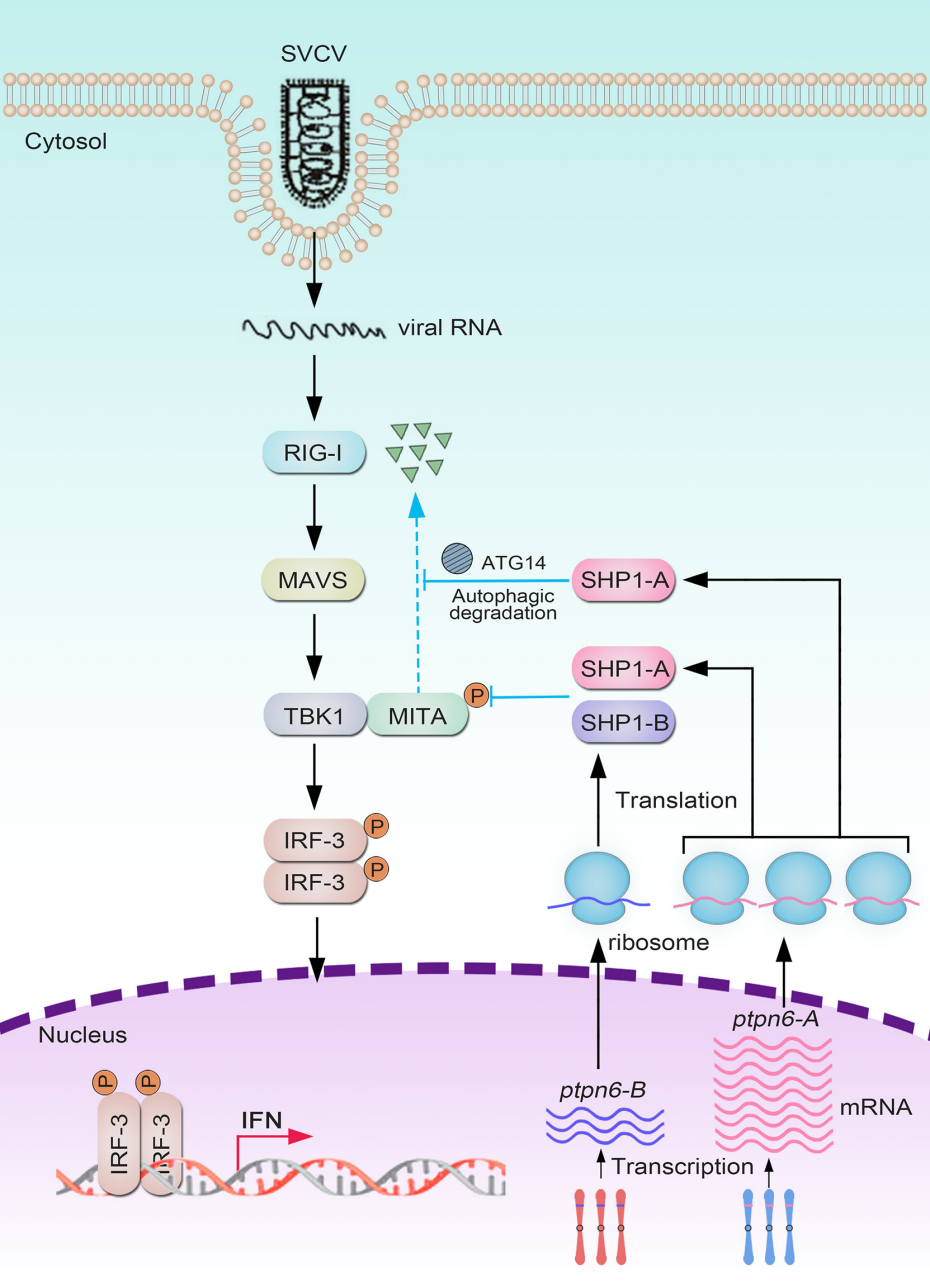
Divergent mechanisms of *Cgptpn6-A* and *Cgptpn6-B* in negatively regulating RLR-mediated signaling pathways.

On the basis of these results, we propose a schematic diagram for the cooperatively and negatively regulative mechanisms of *Cg*SHP1-A and *Cg*SHP1-B in RLR-mediated IFN response (**Figure 9**). In response to SVCV infection, more abundant *Cg*SHP1-A is expressed in relative to *Cg*SHP1-B. In addition to the inhibition of *Ca*TBK1-induced phosphorylation of *Ca*MITA shared with *Cg*SHP1-A and *Cg*SHP1-B, *Cg*SHP1-A also interacts with *Ca*MITA and triggers autophagic degradation of *Ca*MITA. In the fight between fish and aquatic viruses, the ability to mount a properly strong immune reaction is crucial for host survival and health (97, 98). The current findings support that fish SHP1 acts as a negative regulator of RLR-mediated IFN response, which not only sheds light on the functions and regulative mechanism of fish SHP1, but also provides a target gene to breed gibel carp with higher disease-resistance through CRISPR/Cas9 editing. Meanwhile, the above data also provide a typical case of homeolog/allele diversification, biased expression, and sub-functionalization in the evolution of duplicated genes.

## Data Availability Statement

The original contributions presented in the study are included in the article/**Supplementary Material**. Further inquiries can be directed to the corresponding authors.

## Ethics Statement 

The animal study was reviewed and approved by the Institutional Animal Care and Use Committee of IHB, CAS (protocol number 2016-018).

## Author Contributions

J-FG, YW, LZ, and J-FT designed the study. J-FT, SL, L-FL, Z-CL, ZL, R-HG, C-YM, Q-YZ, Z-WW, and X-JZ prepared the samples and carried out the experiments. J-FT, LZ, J-FG, YW, and SL analyzed and discussed the results. LZ, J-FG, SL, YW, and J-FT wrote the paper. All authors contributed to the article and approved the submitted version.

## Funding

This work was supported by the Strategic Priority Research Program of the Chinese Academy of Sciences (XDB31000000, XDA24030203 and XDA24030104), the National Natural Science Foundation (31930111) and China Agriculture Research System of MOF and MARA. The funding bodies had no role in the design of the study and collection, analysis, and interpretation of data and in writing the manuscript.

## Conflict of Interest

The authors declare that the research was conducted in the absence of any commercial or financial relationships that could be construed as a potential conflict of interest.

## Publisher’s Note

All claims expressed in this article are solely those of the authors and do not necessarily represent those of their affiliated organizations, or those of the publisher, the editors and the reviewers. Any product that may be evaluated in this article, or claim that may be made by its manufacturer, is not guaranteed or endorsed by the publisher.

## Supplementary Material

Supplementary Figure 1 Multiple nucleotide sequence alignment of six *Cgptpn6* transcripts from gibel carp clone F. ORF is highlighted by red box **(A)**. Phylogenetic tree of six *Cgptpn6* transcripts in gibel carp **(B)**.

Supplementary Figure 2 Multiple amino acid sequence alignment of *Cg*SHP1 from gibel carp clone F with other vertebrate SHP1 proteins **(A)**. Phylogenetic tree of SHP1 proteins in vertebrates **(B)**.

Supplementary Table 1 Primers used in this study.
