# Supplementary figures and images for: Two Duplicated Ptpn6 Homeologs Cooperatively and Negatively Regulate RLR-Mediated IFN Response in Hexaploid Gibel Carp
Source: Front Immunol. 2021 Nov 26;12:780667. doi: 10.3389/fimmu.2021.780667 (PMC8662705; doi:10.3389/fimmu.2021.780667)

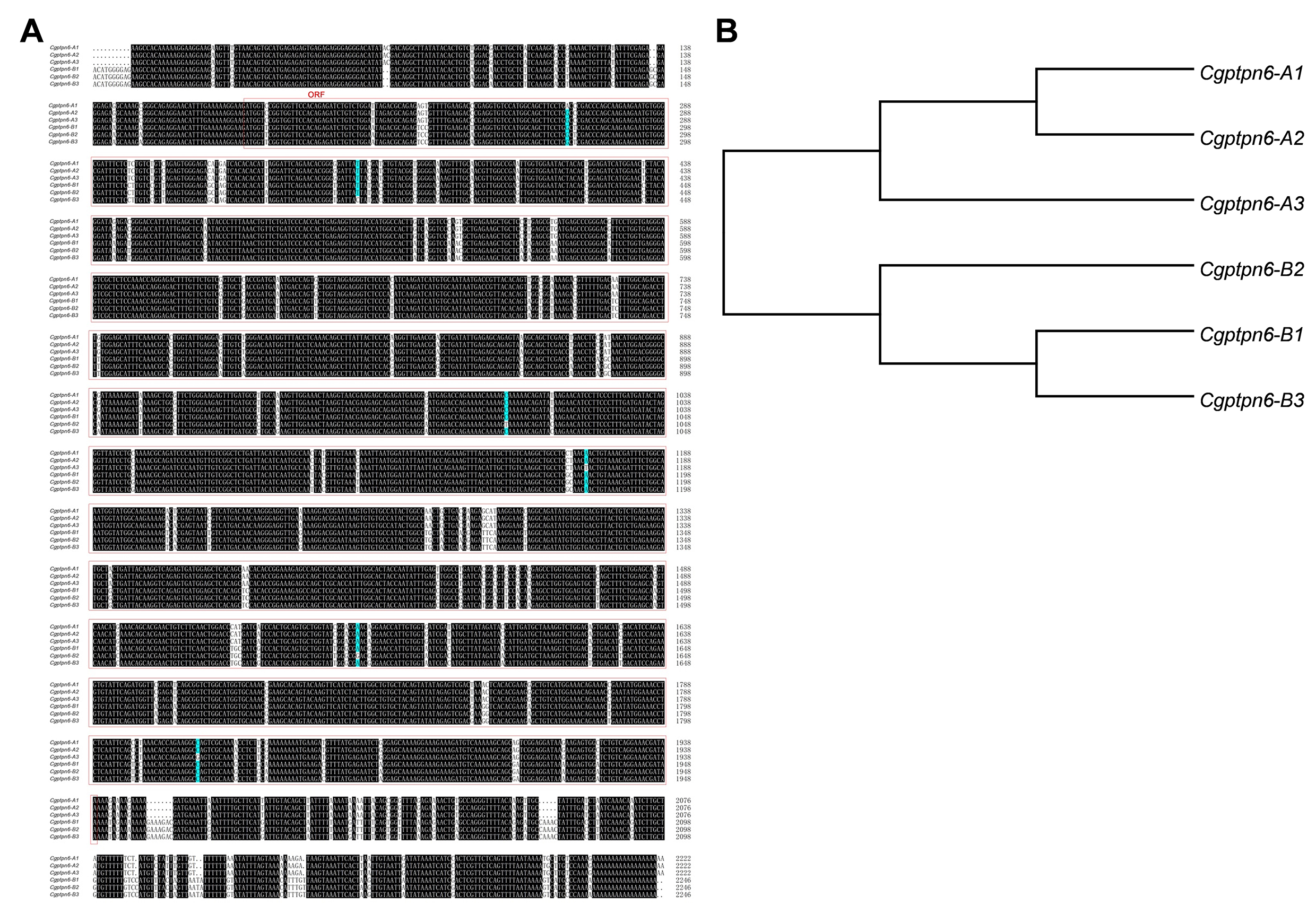

Supplement: Supplementary Figure 1 — Multiple nucleotide sequence alignment of six Cgptpn6 transcripts from gibel carp clone F. ORF is highlighted by red box (A). Phylogenetic tree of six Cgptpn6 transcripts in gibel carp (B). [file Image_1.tif]

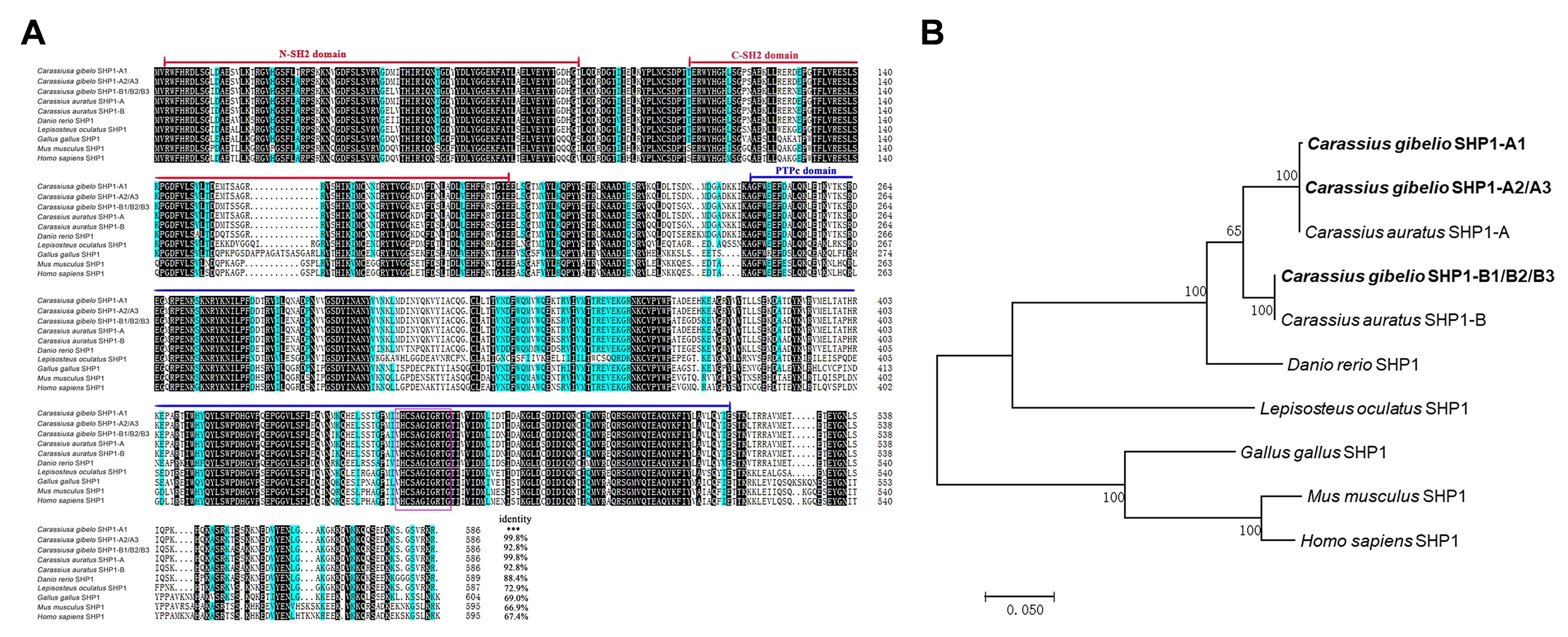

Supplement: Supplementary Figure 2 — Multiple amino acid sequence alignment of CgSHP1 from gibel carp clone F with other vertebrate SHP1 proteins (A). Phylogenetic tree of SHP1 proteins in vertebrates (B). [file Image_2.tif]
